# Performance of the eazyplex® BloodScreen GN as a simple and rapid molecular test for identification of Gram-negative bacteria from positive blood cultures

**DOI:** 10.1007/s10096-021-04383-3

**Published:** 2021-11-22

**Authors:** Katharina Bach, Birgit Edel, Steffen Höring, Lucie Bartoničkova, Stefan Glöckner, Bettina Löffler, Christina Bahrs, Jürgen Rödel

**Affiliations:** 1 Institute of Medical Microbiology, Jena University Hospital, Friedrich Schiller University, Jena, Germany; 2 Division of Infection Control and Infectious Diseases, RWTH Aachen University Hospital, Aachen, Germany; 3 Institute for Infectious Diseases and Infection Control, Jena University Hospital, Friedrich Schiller University, Jena, Germany; 4 Division of Infectious Diseases and Tropical Medicine, Department of Medicine I, Medical University of Vienna, Vienna, Austria

**Keywords:** Gram-negative bacilli, Sepsis, Blood culture, Rapid diagnostics, LAMP

## Abstract

The LAMP-based eazyplex® BloodScreen GN was evaluated for the detection of frequent Gram-negatives directly from positive blood culture (BC) bottles. A total of 449 BCs were analyzed. Sensitivities and specificities were 100% and 100% for *Escherichia coli*, 95.7% and 100% for *Klebsiella pneumoniae*, 100% and 100% for *bla*_CTX-M_, 100% and 100% for *Klebsiella oxytoca*, 100% and 99% for *Proteus mirabilis*, and 100% and 99.8% for *Pseudomonas aeruginosa*, respectively. The time to result ranged from 8 to 16 min, plus about 6 min for sample preparation. The eazyplex® BloodScreen GN is a reliable molecular assay for rapid BC testing.

Sepsis is one of the leading causes of death in the inpatient setting, and appropriate antimicrobial therapy needs to be started in a timely manner [1, 2]. For empiric treatment, the Surviving Sepsis Campaign guideline recommends the administration of intravenous broad-spectrum antibiotics within 1 h following the diagnosis of sepsis or septic shock [3]. Recommendations also include the early targeting of antibiotics when microbiological diagnostic results are available in order to improve the clinical outcome and reduce the selective pressure for resistances [3]. Identification of bloodstream infections (BSI) still relies on blood cultures (BCs). Conventional diagnosis of positive BCs by subcultures and phenotypic antimicrobial susceptibility testing (AST) takes time and results in delays of more than 1 or 2 days before species identification and AST results are reported. MALDI-TOF mass spectrometry (MS) and molecular diagnostic assays can be used to identify the bacterial species and resistance markers directly from a positive BC bottle and are important add-on methods that allow for a more rapid optimization of antibiotic therapy [4–6]. Infectious Disease Society of America (IDSA) guidelines therefore recommend the use of rapid diagnostic tests (RDTs) and their integration in antibiotic stewardship programs [7]. Molecular RDTs based on fully automated random-access multiplex PCRs are very expensive. Loop-mediated isothermal amplification (LAMP) technologies offer the potential to implement fast, simple, and cost-effective molecular tools into diagnostic workflows [8, 9]. Here we evaluated the performance of the CE-labeled LAMP assay eazyplex® BloodScreen GN (Amplex Diagnostics, Gars-Bahnhof, Germany) for identification of frequent Gram-negatives from positive BCs. The assay is based on a previous in-house assay now including additional species-specific primers instead of the ubiquitous target for *Enterobacterales* [9]. A test strip contains ready to use lyophilized master mixes with primers for *Escherichia coli*, *Klebsiella pneumoniae*, *bla*_CTX-M-1 group_, *bla*_CTX-M-9 group_, *Klebsiella oxytoca*, *Proteus mirabilis*, and *Pseudomonas aeruginosa*, and an inhibition control.

Clinical samples were BCs submitted as part of routine patient care from the Jena University Hospital, the SHK Weimar, and the Waldkliniken Eisenberg, to the clinical microbiology laboratory at the Jena University Hospital between September 2019 and May 2020. Blood samples collected in BD BACTEC Plus aerobic/F and lytic/10 anaerobic/F bottles (BD Diagnostics, Heidelberg, Germany) were incubated on a BACTEC FX instrument (BD Diagnostics). An aliquot of positive BCs that revealed Gram-negative rods after Gram-staining was examined by eazyplex® BloodScreen GN and conventional diagnostics in parallel. Only the first positive BC bottle per patient, regardless of whether aerobic or anaerobic, was tested.

For LAMP testing, 5 µl of BC broth was mixed with 500 µl of resuspension and lysis fluid (RALF) and boiled for 2 min. After centrifugation at 4000 rpm for 1 min, 25 µl of the supernatant was added to each tube of the eazyplex^®^ BloodScreen GN test strip. Tests were run on a Genie HT machine (Amplex Diagnostics) at 65 °C for 20 min. Amplification was measured by real-time fluorescence detection using a DNA intercalating dye. Data interpretation was automatically performed by the integrated eazyReport™ software (Amplex Diagnostics). Results are reported as positive in real time if the fluorescence level and the peak of the first derivative of the fluorescence curve rise above the defined thresholds.

For conventional diagnostics, BC aliquots were streaked onto Columbia sheep blood agar, chocolate agar, Schaedler agar (BD), and Drigalski lactose agar (Oxoid, Thermo Fisher Scientific, Wesel, Germany) for overnight incubation at 37 °C. Colonies were identified by Vitek MS (bioMérieux, Nürtingen, Germany). AST was performed by the determination of minimal inhibitory concentrations (MIC) using Vitek 2 (bioMérieux). Breakpoints were interpreted according to European Committee on Antimicrobial Susceptibility Testing (EUCAST) criteria. The production of extended-spectrum beta-lactamases (ESBLs) was verified by the chromogenic β LACTA™ test (Bio-Rad, Feldkirchen, Germany).

A total of 449 positive BCs representing all isolates during the study period were prospectively analyzed. The mean time to positivity of BC bottles, defined as the time between the start of incubation and the positive signal, was 12 (SD, 12.5) h. For the eazyplex® assay, preparation of one sample or two samples in parallel took 5.75 (SD, 0.75; *n* = 6) and 7 (SD, 0.75; *n* = 6) min, respectively. The time to result, defined by the threshold time of fluorescence intensity, ranged from about 8 min for *P. mirabilis* to 16 min for *K. pneumoniae* (Table 1). For 20 samples, the inhibition control of the assay was invalid. They were therefore excluded from the evaluation (4.5% of all BCs). The eazyplex® BloodScreen GN demonstrated high sensitivity and specificity for all targets (Table 2). All *E. coli* and *K. pneumoniae* cases with a positive CTX-M result were phenotypically confirmed as ESBL-producing isolates by AST (Table 3). ESBL-producing but CTX-M-negative isolates were not detected. There were two false-negative and four false-positive results in regard to the species-specific targets. The false positives were observed for *P. mirabilis* caused by cross-reactions with *E. coli*. Two false-negative results were attributed to *K. pneumoniae* and *K. variicola* as identified by Vitek MS. It should be noted that the assay cannot differentiate between both *Klebsiella* species (information by the manufacturer). From 79 BCs with a valid inhibition control, no species-specific test results were obtained (18.4%). Subculture identification revealed as most frequent pathogens *Enterobacter cloacae* complex (*n* = 18), *Bacteroides fragilis* (*n* = 12), *Serratia marcescens* (*n* = 6), non-typhoidal *Salmonella enterica* (*n* = 6), *Citrobacter freundii* complex (*n* = 5), and *Acinetobacter baumannii* complex (*n* = 3). Mixed infections with Gram-negatives or Gram-positives were detected in 23 BCs. In all cases, the species included in the eazyplex® assay were correctly identified. In 7 out of 10 cases of a mixed Gram-negative infection, both species were covered by the assay.

**Table 1:** Time to result of the eazyplex^®^ BloodScreen GN assay

eazyplex®	Threshold time (min; mean values (SD))
*E. coli*	8.5 (1.75)
*bla*_CTX-M-1 group_ (*E. coli*)	6.5 (2.25)
*bla*_CTX-M-9 group_ (*E. coli*)	8 (2.25)
*K. pneumoniae*	15.75 (2.25)
*bla*_CTX-M-1 group_ (*K. pneumoniae*)	6.5 (0.75)
*K. oxytoca*	15 (3.25)
*P. mirabilis*	7.75 (1.75)
*P. aeruginosa*	9 (0.75)
Inhibition control	11.25 (1)

**Table 2:** Performance of the eazyplex^®^ BloodScreen GN assay for BCs^a^

eazyplex® target	True positive (*n*)	True negative (*n*)	False positive (*n*)	False negative (*n*)	Sensitivity, % (CI^b^)	Specificity, % (CI^b^)	PPV, %^c^ (CI^b^)	NPV, %^d^ (CI^b^)
*E. coli*	248	181	0	0	100 (98.5–100)	100 (98–100)	100	100
CTX-M^e,g^ (*E. coli*)	42	206	0	0	100 (91.6–100)	100 (98.2–100)	100	100
*K. pneumoniae*	42	385	0	2	95.5 (84.5–99.4)	100 (99.1–100)	100	99.5 (98.2–99.9)
CTX-M^f,g^ (*K. pneumoniae*)	9	35	0	0	100 (66.4–100)	100 (90–100)	100	100
*K. oxytoca*	23	406	0	0	100 (86.2–100)	100 (99.1–100)	100	100
*P. mirabilis*	22	403	4	0	100 (84.6–100)	99 (97.5–99.7)	84.6 (67.5–93.6)	100
*P. aeruginosa*	22	406	1	0	100 (84.6–100)	99.8 (98.6–100)	95.7 (75.7–99.4)	100

^a^Data from 429 BCs were analyzed. Only one positive bottle per patient was tested. ^b^*CI*, 95% confidence interval; ^c^*PPV*, positive predictive value; ^d^*NPV*, negative predictive value; ^d^*bla*_CTX-M-1 group_: *n* = 35, *bla*_CTX-M-9 group_: *n* = 7; ^f^*bla*_CTX-M-1 group_: *n* = 9; ^g^As markers for ESBL production, the CTX-M results were defined as true positive or true negative when an ESBL phenotype was identified or ruled out, respectively

**Table 3:** Comparison of eazyplex® results with antibiotic resistance patterns of BC isolates

eazyplex® result	Antibiotic resistance, resistant/total (%)				
	Piperacillin-tazobactam	Cefotaxime	Ceftazidime	Meropenem	Ciprofloxacin
*E. coli*, CTX-M negative	11/206 (5.3)	0/206 (0)	0/206 (0)	0/206 (0)	32/206 (15.5)
*E. coli*, CTX-M positive	5/42 (11.9)	42/42 (100)	42/42 (100)	0/42 (0)	25/42 (59.5)
*K. pneumoniae*, CTX-M negative	3/35 (9.4)	0/35 (0)	0/35 (0)	0/35 (0)	2/35 (5.7)
*K. pneumoniae*, CTX-M positive	6/9 (66.7)	9/9 (100)	9/9 (100)	0/9 (0)	5/9 (55.6)
*K. oxytoca*	2/23 (8.6)	1/23 (4.3)	1/23 (4.3)	0/23 (0)	5/23 (21.7)
*P. mirabilis*	0/22 (0)	0/22 (0)	0/22 (0)	0/22 (0)	3/22 (13.6)
*P. aeruginosa*	7/22 (33.8)	NA^a^	6/22 (27.3)	3/22 (13.6)	5/22 (22.7)

^a^*NA*, not applicable

At the Jena University Hospital, piperacillin-tazobactam is primarily recommended for empiric antibiotic treatment of sepsis in patients with no documentation of prior infection or colonization with MRSA or multidrug-resistant Gram-negatives. Although the majority of ESBL-producing *E. coli* isolates were sensitive against piperacillin-tazobactam, escalation to meropenem based on a positive eazyplex® CTX-M result would be appropriate (Table 3). Several studies have shown that piperacillin-tazobactam appears to be inferior to carbapenems for treatment of serious infections and bacteremia caused by ESBL-producing organisms, underlining the potential benefit of RDTs to identify ESBL resistance 1 day earlier than conventional phenotypic AST [10–12].

De-escalation based on eazyplex® results without knowledge of phenotypic AST is unsafe. According to the ABS recommendations at our hospital, identification of CTX-M-negative *E. coli* and *P. mirabilis* would result in continuation of piperacillin-tazobactam, whereas non-ESBL *K. pneumoniae* and *K. oxytoca* are preferentially treated with a third-generation cephalosporin (Table 3) [4]. Identification of *P. aeruginosa* would require optimization of piperacillin-tazobactam dosing but would not allow early targeting of antibiotics unless diagnostic results and resistance information from other relevant samples are available (Table 3). When the eazyplex® assay shows no species-specific result, the presence of a SPICE organism (*Serratia* spp., *Pseudomonas* spp., Indole-positive *Proteus* group, *Citrobacter* spp., and *Enterobacter* spp.) or Acinetobacter spp. can be expected in most of the cases and escalation of the therapy to meropenem may be considered at least until the species is identified [13, 14].

Clinical studies on the impact of rapid BC testing on patient outcome could demonstrate that the mortality risk and length of stay decreased when a molecular RDT is implemented in ABS [15–17]. The results of this study show that the eazyplex® BloodScreen GN is a viable rapid assay with little workload. A further advantage of the assay is the relatively low cost for consumables (about 35 EUR per test) compared to automated random-access PCR assays that can be more than twice as expensive. MALDI-TOF MS analysis directly performed on BC aliquots has low consumable costs but is more labor-intensive due to the need for centrifugation steps and additional testing for beta-lactamases [18]. Additional costs of rapid BC testing must be balanced against the expected clinical benefit [19, 20]. RDT results more likely lead to escalation, whereas de-escalation will be rather considered when final AST results are available. In this context, it must be considered that the eazyplex® assay covers resistances against third-generation cephalosporins only by detection of *bla*_CTX-M_. Unfortunately, for this assay, no appropriate LAMP primer target that covers all species of the *E. cloacae* complex could be found (information by the manufacturer). Upregulated AmpC beta-lactamases in these species are responsible for inactivating third-generation cephalosporins [21, 22]. The lack of these targets may be also problematic in mixed infections [23]. Of note is the availability of eazyplex® carbapenemase test kits that can be performed as additional tools depending on the local prevalence of carbapenemase-producing Gram-negatives or the colonization status of the patient [24].

The eazyplex® BloodScreen GN has now been implemented in routine diagnostics in our laboratory. Test results are immediately reported to the clinician and/or infectiologist. Whether the ongoing antibiotic therapy is continued or escalated based on the eazyplex® result depends on additional diagnostics reports available for the patient. A treatment algorithm, which takes both the eazyplex® result and the hospital-specific antibiogram into account, has been developed (Table 4). In the context of this study, the proposed algorithm would have resulted in a recommendation of meropenem in 30% of all cases. In 22% of all cases, the use of meropenem would have been appropriate because of the isolation of *Acinetobacter* spp. (*n* = 6), ESBL-producers (*n* = 51), and SPICE organisms excluding *P. aeruginosa* (*n* = 38) from BCs.

**Table 4:** Antibiotic treatment recommendations for the treatment of BSI caused by Gram-negatives identified by the eazyplex® BloodScreen GN^a^

eazyplex® result	Preferred antibiotic^b^
*E. coli*, CTX-M negative	Piperacillin-tazobactam
*E. coli*, CTX-M positive	Meropenem
*K. pneumoniae*, CTX-M negative	Ceftriaxone or cefotaxime
*K. pneumoniae*, CTX-M positive	Meropenem
*K. oxytoca*	Ceftriaxone or cefotaxime
*P. mirabilis*	Piperacillin-tazobactam
*P. aeruginosa*	High-dose piperacillin-tazobactam or high-dose ceftazidime
Negative	Meropenem

^a^These recommendations consider the annual hospital antibiogram. ^b^De-escalation may be indicated as soon as the definitive antibiogram is available. Consultation with the infectious disease service at the hospital should be considered

In conclusion, the eazyplex® BloodScreen GN is a simple diagnostic tool for rapid BC testing. Because of the high accuracy in the identification of species and CTX-M genes, results can be used for a timely escalation of empiric therapy when indicated.

## Data Availability

The dataset analyzed in this study is available from the corresponding author on reasonable request.
